# Transient expansion of peripheral Lambda-expressing plasma cells represents a distinctive phenotype associated with SFTSV infection

**DOI:** 10.3389/fimmu.2026.1763231

**Published:** 2026-04-24

**Authors:** Yawei Tang, Jingxue Wu, Yao Tian, Jiao Wang, Jianwei Liu, Qingyan Chang, Jie Zhu

**Affiliations:** 1 Department of Flow Cytometry Center, the Second Hospital of Dalian Medical University, Dalian, China; 2 Pharmacy Department, Dalian Public Health Clinical Center, Dalian, China

**Keywords:** Severe fever with thrombocytopenia syndrome, SFTS infection, Lambda-expressing plasma cells, distinctive immunophenotyping, diagnosis

## Abstract

**Objective:**

Severe fever with thrombocytopenia syndrome (SFTS) is an emerging infectious disease with a high fatality rate. The mechanism of SFTS is widely recognized to be closely associated with dysregulated host immune responses. However, comprehensive assessments of peripheral immune patterns in SFTS remains limited. This study aimed to characterize the profiles of peripheral immune responses and identify unique immune subsets involved in the pathogenesis of SFTS.

**Methods:**

Twenty-four patients diagnosed with SFTS were enrolled in this study. Flow cytometric analysis was employed to determine the percentages, absolute counts, and immunophenotypes of immune cells. An external validation cohort of eighteen patients was used to validate the Lambda-expressing plasma cells expansion in SFTS.

**Results:**

Patients with SFTS exhibited markedly increased activation and exhaustion of CD4^+^ and CD8^+^ T cells compared to healthy controls. This was accompanied by a significant expansion of T peripheral helper cells and plasmablasts. Additionally, an enrichment of plasma cells expressing the Lambda light chain was observed in SFTS patients. These Lambda-expressing plasma cells displayed a normal immunophenotype, were transiently present in peripheral blood, and disappeared upon recovery from SFTS virus (SFTSV) infection, distinguishing them from the malignantly expanded plasma cells typically reported in multiple myeloma. Furthermore, the frequency of Lambda-expressing plasma cells was correlated with the clinical severity and outcomes in SFTS patients in the discovery and validation cohorts, respectively.

**Conclusions:**

This study is the first to identify an expansion of peripheral transient Lambda-expressing plasma cells in SFTS cases, representing a distinctive immunological hallmark of SFTSV infection. These findings provide valuable insights into the pathogenesis of SFTS and may facilitate the development of improved diagnostic and therapeutic approaches for patients with SFTS.

## Introduction

Severe fever with thrombocytopenia syndrome (SFTS) is an emerging infectious disease caused by SFTS virus (SFTSV), a novel phlebovirus within the *Bunyaviridae* family (1). First identified in China in 2009, SFTS has been reported in several countries, including Korea, Japan, Vietnam, and Myanmar (2–4). Since its discovery, the incidence of SFTS has increased rapidly, posing significant public health challenges. In 2017, the World Health Organization listed SFTS among the top ten priority infectious diseases (5). Currently, no effective therapeutics or vaccines are available for the treatment or prevention of SFTS.

Although the underlying mechanisms of SFTSV infection are not yet fully understood, it is widely acknowledged that the pathogenesis of SFTS is closely linked to dysregulated host immune responses, which play a key role in disease progression and severity (6, 7). Previous studies have shown that innate immune cells, including monocytes, macrophages, natural killer (NK) cells, and dendritic cells (DC) in peripheral blood are significantly reduced in severe SFTS cases and exhibit a correlation with clinical outcomes (8, 9). Additionally, severe or fatal SFTS cases have demonstrated marked depletion of CD3^+^, CD4^+^ T cells, alongside an increased proportion of regulatory T cells (10, 11). Emerging evidence indicates that SFTSV can directly infect B-cell lineage populations, identifying B cells as critical targets of lethal SFTSV infection (12, 13). Postmortem analyses of lymph nodes have confirmed that the majority of SFTSV-infected cells are B cells, particularly plasmablasts and plasma cells (14). Moreover, high viral loads, hyperinflammatory responses, and abnormal complement activation have been observed in fatal SFTS cases (15). While these clinical and immunological findings highlight the pivotal role of immune cells in the pathogenic progression of SFTS, a comprehensive assessment of human peripheral immune responses remains limited. To further elucidate the relationship between viral pathogenesis and host immunity, a deeper understanding of the immune landscape in SFTS is essential, which could provide critical guidance for the timely and effective intervention of SFTSV infection.

As a novel tick-borne disease, SFTS primarily affects farmers residing in wooded and hilly regions (16). Many patients diagnosed with SFTS report a history of tick bites prior to the onset of symptoms (17). The major clinical manifestations of SFTS include the rapid onset of high fever, gastrointestinal symptoms, and general fatigue during the early phase, followed by deterioration of consciousness and hemorrhagic tendencies in the later stages (18, 19). Leukopenia, thrombocytopenia, and elevated liver-related aminotransferase are the most common abnormalities in laboratory findings in SFTS cases (20). However, these clinical and laboratory features are nonspecific. Several diseases, such as leptospirosis, hemorrhagic fever with renal syndrome (HFRS), human granulocytic anaplasmosis, dengue fever, and typhoid fever, primary immune thrombocytopenia, and aplastic anemia (AA), share overlapping clinical presentations with SFTS (21–23). This diagnostic overlap increases the risk of misdiagnosis, potentially raising mortality rates and adversely affecting outcomes of patients with SFTS. While virus isolation from blood is a reliable diagnostic method for SFTSV infection, it is time-consuming and requires high-security biocontainment facilities (24). Several molecular techniques, including reverse-transcription polymerase chain reaction (RT-PCR), quantitative real-time RT-PCR, and reverse transcription loop-mediated isothermal amplification assay (RT-LAMP), have been developed to detect the SFTSV genome (25, 26). However, these methods are most effective only during the acute phase of infection, typically within 1–6 days after symptom onset (27). Therefore, the development of reliable, rapid, sensitive, and specific laboratory diagnostic methods capable of differentiating SFTSV infection from febrile and thrombocytopenia-associated diseases is essential, which would help clinicians to make timely and accurate diagnoses.

In this study, we provide a comprehensive characterization of the immune landscape in a cohort of 24 hospitalized patients diagnosed with SFTSV infection. Notably, our findings revealed that the overproliferation of circulating Lambda-expressing plasma cells serves as a distinctive immunological hallmark of SFTSV infection. Based on the flow cytometric features of these peripheral plasma cells, we developed a flowchart for early diagnosis of SFTS. Furthermore, the frequency of Lambda-expressing plasma cells exhibited a correlation with the clinical severity and prognosis in SFTS patients, which were further confirmed by an independent external cohort. Collectively, this systematic analysis highlights that SFTSV infection induces expansion of Lambda-expressing plasma cells in peripheral blood, enriching our understanding of viral pathogenesis and offering valuable insights into the diagnosis and potential treatment of SFTS patients.

## Methods

### Study design and participants

A control cohort study was conducted to analyze the change of peripheral immune responses in SFTS patients. Between May 2021 and July 2024, 24 patients diagnosed with SFTSV infection and admitted to the Second Affiliated Hospital of Dalian Medical University were included. Besides, an independent validation cohort comprising 18 patients with SFTS admitted to the Dalian Public Health Clinical Center between October 2024 and October 2025. The diagnosis of SFTSV infection was based on the guidelines issued by the National Health Commission of China (28). Patients were excluded if (1) aged<18 years; (2) infected with hepatitis C virus (HCV), hepatitis B virus (HBV), or human immunodeficiency virus (HIV)-1. Additionally, 20 healthy controls (HC) were recruited during the study period to serve as normal controls. These controls were selected from individuals undergoing healthy physical examinations aged≥18 years and without HCV, HBV, or HIV-1 infections.

An additional cohort study was performed to compare the immunophenotype and number of plasma cells between patients with SFTS and those with SFTS-like diseases, characterized by febrile symptoms or thrombocytopenia. A total of 67 patients were enrolled at the Second Affiliated Hospital of Dalian Medical University between June 2023 and July 2024. This cohort included 10 patients with brucellosis, 12 patients with severe pneumonia, 8 patients with sepsis, 2 patients with Epstein-Barr virus (EBV) infection, 1 patients with scrub typhus, 2 patients with HFRS, 10 patients with rheumatoid arthritis (RA), 4 patients with systemic lupus erythematosus (SLE), 3 patients with idiopathic thrombocytopenic purpura (ITP), 9 patients with megaloblastic anemia (MA), 1 patients with AA, and 5 patients with myelodysplastic syndrome (MDS). Demographic information, including gender, age, and clinical parameters for these patients is presented in **Supplementary Data Sheet 2**.

Peripheral blood samples were collected from all participants within 24 hours of admission. Additionally, bone marrow examinations were performed on 9 patients diagnosed with SFTS during their hospitalization, and bone marrow samples were obtained from these individuals. All samples were anticoagulated with ethylene diamine tetraacetic acid and processed within 4 hours of collection. This study was approval by the Ethics Committee of the Second Affiliated Hospital of Dalian Medical University and Ethics Committee of the Dalian Public Health Clinical Center.

### Definitions

SFTS patients were classified into mild and severe groups based on clinical symptoms and disease severity. The diagnostic criteria of mild and severe SFTS patients were determined according to the Guidelines for the Diagnosis and Treatment of Severe Fever with Thrombocytopenia Syndrome issued by the National Health Commission of the People’s Republic of China (29). The criteria for inclusion in the mild group were: (1) the body temperature <39°C; (2) the patients showed fatigue, anorexia, nausea, vomiting, or diarrhea symptoms, (3) the patients no altered mental status, obvious bleeding tendency, or organ dysfunction; (4) One or more laboratory findings of the following: ① platelet count ≥50×10^9^/L; ② the levels of alanine aminotransferase (ALT), aspartate aminotransferase (AST), or lactate dehydrogenase (LDH) no more than three fold the upper limit of normal. The criteria for inclusion in the severe group were: (1) the body temperature ≥39°C; (2) the patients showed central nervous system involvement (drowsiness, restlessness, delirium, or coma), overt bleeding manifestations, or multiple organ dysfunction; (3) One or more laboratory findings of the following: ① platelet count <50×10^9^/L; ② the levels of ALT, AST, or LDH were more than three fold the upper limit of normal.

### Clinical data collection

Demographic and clinical data of SFTS patient were collected from electronic and paper medical records, including the following variables: (1) age and sex; (2) underlying diseases; (3) clinical symptoms; (4) laboratory results, such as white blood cell (WBC) count, red blood cell (RBC) count, monocyte count, lymphocyte count, platelet (PLT), hemoglobin (HGB), AST, ALT, albumin (ALB), LDH, cardiac troponin I (cTnI), creatine kinase isoenzyme (CK-MB), fibrinogen, D-dimer, activated partial thromboplastin time (APTT), prothrombin time (PT), thrombin time (TT), C-reactive protein (CRP), procalcitonin (PCT), IL-2, IL-4, IL-6, IL-10, IFN-γ, and TNF-α; (5) treatment regimens; (6) outcomes: the primary outcome was survival or death, which data was retrieved from medical records or obtained by following up the patients who discontinued, with a cut-off point of 28 days.

### Absolute lymphocyte count in peripheral blood

The percentages and absolute counts of CD3^+^, CD4^+^, CD8^+^ T cells, and CD3^-^CD56^+^ NK cells in peripheral blood were determined using TruCount tubes and the BD Multitest 4-color TBNK Reagent Kit (BD Biosciences, USA) according to the manufacturer’s instructions. Each blood sample was processed in two tubes containing fluorescent monoclonal antibodies (mAb). One tube contained CD3-FITC, CD8-PE, CD45-PerCp, and CD4-APC, while the other tube contained CD3-FITC, CD16-PE, CD56-PE, CD45-PerCp, and CD19-APC. For lymphocyte subset detection, 40 μL of whole blood was added to the tubes and mixed with 20 μL of the indicated mAb. The tubes were vortexed gently and incubated in the dark at room temperature for 15 minutes. Subsequently, 370 μL of diluted Red Blood Cell Lysis Buffer (BD Biosciences, USA) was added and mixed, and the tubes were kept in the dark at room temperature for an additional 15 minutes. Samples were sorted and analyzed on the BriCyte E6 flow cytometer (Mindray, China). For absolute cell count, the total number of fluorescent microbeads was used as the standard internal parameter, and fluorescently labeled antibodies were added to the tubes. Flow cytometry acquisition and analysis software was employed to calculate the cell count using the following formula: cells/μL=(acquired cells×total beads)÷(acquired beads×volume of the sample)×100%.

### Flow cytometry analysis

Flow cytometry analysis was performed to assess the peripheral immune responses in SFTS patients. Briefly, three panels were designed and the following mAb were added to 100 μL of whole blood. The details were as follows: (1) panel 1: anti-CD38-FITC, anti-PD-1-PE, anti-CD3-Percp, anti-HLA-DR-APC, anti-CD4-APC-Cy7, anti-CD8-PE-Cy7, anti-CD56-BV421, anti-CD45-BV510; (2) panel 2: anti-CD27-FITC, anti-PD-1-PE, anti-CD38-Percp, anti-CD24-APC, anti-CD4-APC-Cy7, anti-CD19-PE-Cy7, anti-CXCR5-BV421, anti-CD45-BV510; (3) panel 3: anti-Kappa-FITC, anti-Lambda-PE, anti-CD19-Percp, anti-CD138-APC, anti-CD20-APC-Cy7, anti-CD38-PE-Cy7, anti-CD56-BV421, anti-CD45-BV510. Isotype-matched control antibodies were included as negative controls. Detailed information on the fluorescence-conjugated antibodies is provided in **Supplementary Table 1**. The samples were stained with mAb targeting surface markers for 15 minutes in the dark at room temperature. Intracellular protein staining for cytoplasmic Kappa (cKappa) and cytoplasmic Lambda (cLambda) light chains was performed using the Cytofix/Cytoperm kit (BD biosciences) according to the manufacturer’s instructions. After lysing red blood cells with Red Blood Cell Lysis Buffer, the pellets were washed and resuspended in 500 μL PBS. Flow cytometry was conducted on the BD Canto II flow cytometer, and data were analyzed with FlowJo software (version 10.2).

### Statistical analysis

Normally distributed continuous variables are presented as means ± standard deviation; otherwise, the 25%, median, and 75% places were provided. Categorical variables are presented as percentages. Statistical significance was determined using the Mann-Whitney *U* test, Student’s *t*-test, one-way ANOVA, or the chi-squared test. Spearman correlation was used to examine the relationship between plasma cell subsets in peripheral blood and laboratory characteristics. Statistical analysis was performed using GraphPad Prism (version 8.0) or SPSS (version 18.0). *p* < 0.05 was considered statistically significant.

## Results

### Baseline characteristics of SFTS patients

**Figure 1:**
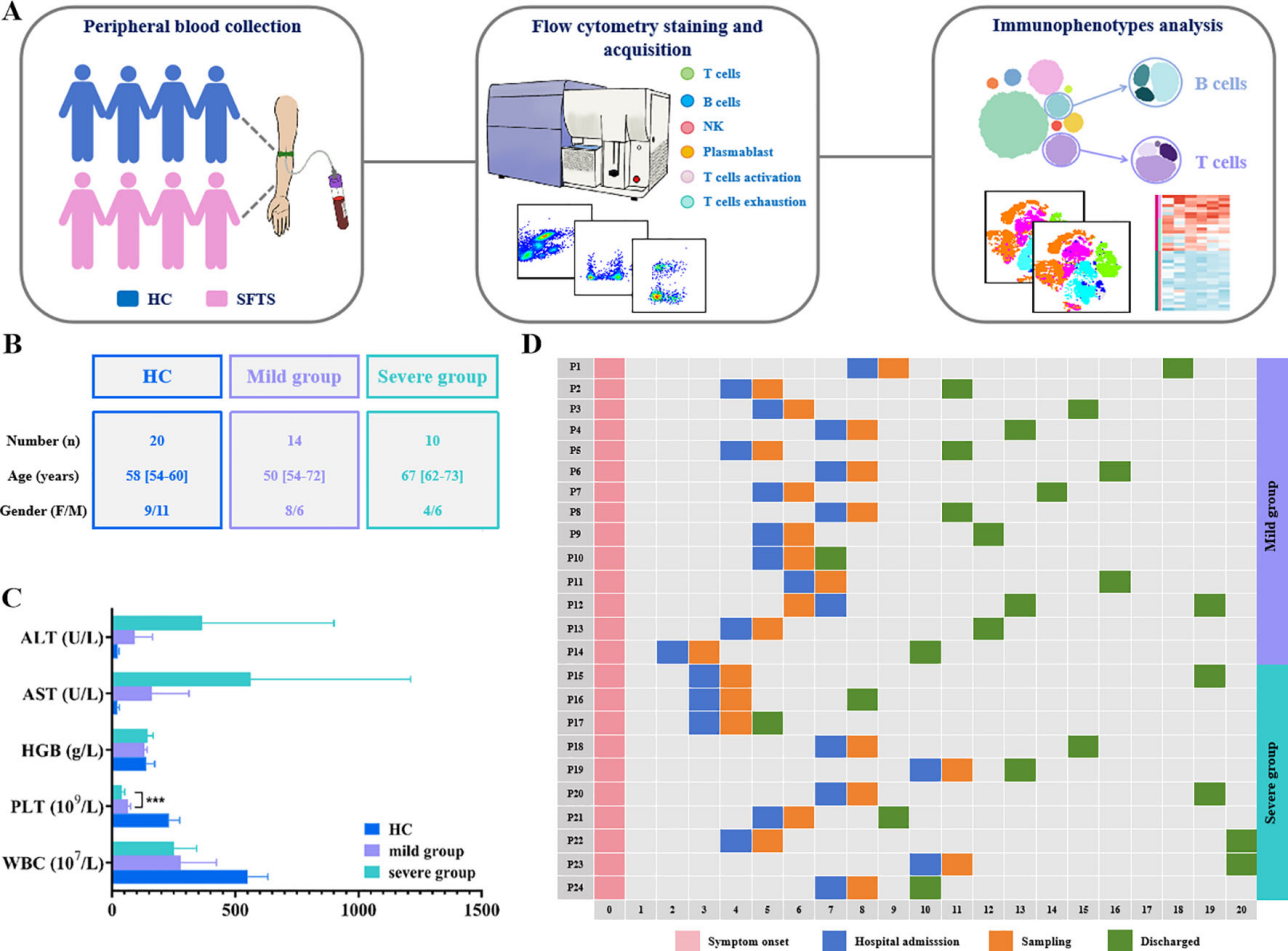
Graphical overview of SFTS patients and HC enrolled in the study. **(A)** Overview of the study workflow. **(B, C)**. Demographics and clinical characteristics of SFTS patients and HC. **(D)** Disease course timeline for 24 patients with SFTS enrolled in the study.

A total of 24 patients diagnosed with SFTS were enrolled in the study, including 12 females and 12 males, with a median age of 66 years (interquartile range, 57–72 years). SFTS patients were divided into a mild group (n=14) and a severe group (n=10) based on the clinical symptoms and disease severity at admission. Additionally, 20 age- and sex-matched HC (11 females and 9 males), with a median age of 58 years (interquartile range, 54–60 years) were also included (**Figure 1**). Compared to the mild group, patients in the severe group exhibited significantly elevated PLT count, LDH concentration, APPT, TT, D-dimer, and CRP level. There were no statistically significant differences between the mild and severe groups in other clinical parameters, including age, sex, WBC count, lymphocyte count, AST level, ALT level, CK-MB level, and PCT level. Detailed demographic and clinical characteristics of SFTS patients is presented in **Table 1** and **Supplementary Data Sheet 1**.

**Table 1:** Demographics and basic laboratory characteristics of SFTS patients.

Characteristics	All patients (n=24)	Mild group (n=14)	Severe group (n=10)	*P* value
Demographics feature				
Age (years)	66 (57, 72)	50 (54, 72)	67 (62, 73)	0.333
Gender				
Male, n (%)	12 (50%)	6 (43%)	6 (60%)	0.408
Female, n (%)	12 (50%)	8 (57%)	4 (40%)	
Blood routine tests				
WBC (10^9^/L)	2.59 (1.73, 3.22)	2.62 (1.52, 3.16)	2.4 (1.8, 3.3)	0.977
RBC (10^12^/L)	4.50 (4.15, 5.02)	4.42 (4.22, 4.61)	4.80 (4.10, 5.23)	0.412
Monocyte (10^9^/L)	0.09 (0.05, 0.34)	0.14 (0.06, 0.47)	0.08 (0.04, 0.14)	0.290
Lymphocyte (10^9^/L)	0.69 (0.41, 0.99)	0.97 (0.45, 1.17)	0.56 (0.53, 0.93)	0.241
PLT (10^9^/L)	57 (38, 66)	65 (58, 72)	37 (33,45)	<0.001
HGB (g/L)	135 (123, 151)	131 (123, 138)	151 (117, 163)	0.177
Chemistry tests				
AST (U/L)	158 (72, 348)	118 (66, 198)	300 (141, 986)	0.069
ALT (U/L)	84.51 (42.18, 186.68)	58.86 (41.26, 144.30)	139.66 (79.87, 524.31)	0.079
ALB (g/L)	30.27 (27.82, 34.90)	32.37 (27.86, 36.22)	30.10 (27.73, 32.35)	0.482
LDH (U/L)	666.33 (478.82, 1317.45)	488.51 (469.83, 705.23)	1309.54 (819.15, 2165.18)	0.006
cTnI (mg/L)	0.05 (0.02, 0.07)	0.03 (0.01, 0.07)	0.06 (0.04, 0.09)	0.095
CK-MB (mg/L)	2.65 (1.48, 4.78)	2.65 (1.40, 5.45)	3.20 (1.93, 4.53)	0.860
Coagulation indicators				
APTT (s)	50.15 (43.18, 60.93)	44.90 (38.43, 50.28)	61.30 (52.70,70.70)	<0.001
PT (s)	12.60 (12.03, 13.48)	12.60 (11.43, 13.25)	12.65 (12.33, 13.63)	0.481
TT (s)	23.85 (20.93, 25.90)	21.65 (20.38, 24.05)	26.95 (23.40, 38.530)	0.002
D-dimer (mg/mL)	1.54 (1.09, 3.00)	1.18 (0.96, 1.68)	2.75 (1.76, 6.28)	0.003
Fibrinogen (g/L)	2.47 (2.03, 2.93)	2.44 (1.98, 3.01)	2.56 (2.04, 2.90)	0.66
Inflammation indicators				
CRP (mg/L)	5.08 (2.54, 12.86)	4.62 (1.99, 8.22)	6.40 (4.48, 25.70)	0.198
PCT (ng/mL)	0.33 (0.16, 0.43)	0.24 (0.11, 0.34)	0.38 (0.28, 0.58)	0.037

Data were shown as median (interquartile range) or n (%). *P* values were calculated by two-sided Mann-Whitney *U* test or chi-square test. WBC, white blood cell, RBC, red blood cell, PLT, platelet, HGB, hemoglobin, AST, aspartate aminotransferase, ALT, alanine aminotransferase, ALB, albumin, LDH, lactate dehydrogenase, cTnI, cardiac troponin I, CK-MB, creatine kinase isoenzyme, APTT, activated partial thromboplastin time, PT, prothrombin time, TT, thrombin time, CRP, C-reactive protein, PCT, procalcitonin.

### Dynamic changes in peripheral T cell activation and exhaustion in SFTS patients

**Figure 2:**
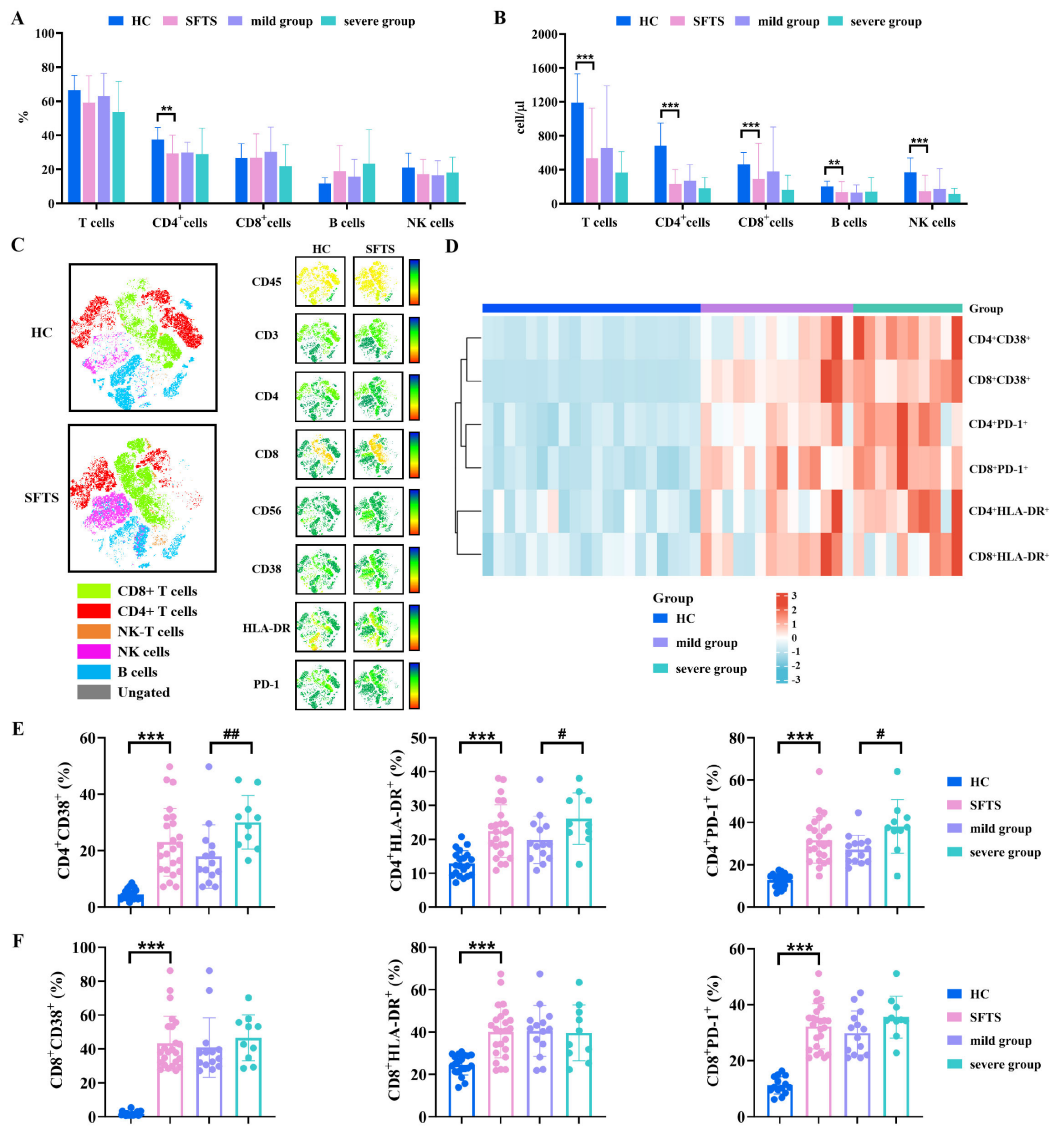
Differences in lymphocyte composition and T cell immune responses between SFTS patients and HC. **(A, B)** Percentages and absolute counts of T cells, B cells, and NK cells in SFTS patients and HC. **(C)** t-SNE map of CD45^+^ lymphocyte cells from SFTS patients and HC. **(D)** Heatmap displaying scaled expression values of the 6 subsets between SFTS patients and HC. **(E, F)** Expression of CD38, HLA-DR, and PD-1 on CD4^+^ and CD8^+^ T cells were analyzed between SFTS patients and HC. *Data were compared between SFTS patients and HC. #Data were compared between mild group and severe group in patients with SFTS. ***p < 0.001, #*p* < 0.05, ##*p* < 0.01.

To gain a comprehensive understanding of the lymphocyte composition in SFTS patients, we performed flow cytometry to analyze the absolute counts and percentages of lymphocyte subsets SFTS patients and HC. As shown in **Figures 2A, B**, the absolute counts of T cells, B cells, and NK cells were significantly reduced in the SFTS group, with a notable decrease in the percentage of CD4^+^ T cells compared to the HC group. Furthermore, we compared lymphocyte subsets between the mild and the severe groups of SFTS patients. No significant differences were observed in the absolute counts and frequencies of lymphocyte subsets between these groups.

Subsequently, to investigate the T cells immune responses during SFTSV infection, we assessed the activation and exhaustion status of CD4^+^ and CD8^+^ T cells by flow cytometry. In this study, we focused on the expression of CD38 and HLA-DR as activation markers, and PD-1 as an exhaustion marker, which together reflect the dynamic balance between immune activation and functional impairment in the process of SFTSV infection. The t-SNE map and hierarchical cluster analysis were performed to visualize the activation and exhaustion markers of T cells in SFTS patients and HC (**Figures 2C, D**). Notably, SFTS patients exhibited significantly higher expression of CD38, HLA-DR, and PD-1 on CD4^+^ and CD8^+^ T cells compared to HC (**Figures 2E, F**). Furthermore, the percentages of CD4^+^CD38^+^, CD4^+^HLA-DR^+^, and CD4^+^PD-1^+^ cells were significantly higher in the severe group compared to the mild group among SFTS patients (**Figure 2E**). However, no significant differences were observed in the frequencies of CD8^+^CD38^+^, CD8^+^HLA-DR^+^ and CD8^+^PD-1^+^ cells between the two groups (**Figure 2F**).

### Altered peripheral T- and B-cell composition in SFTSV infection patients

**Figure 3:**
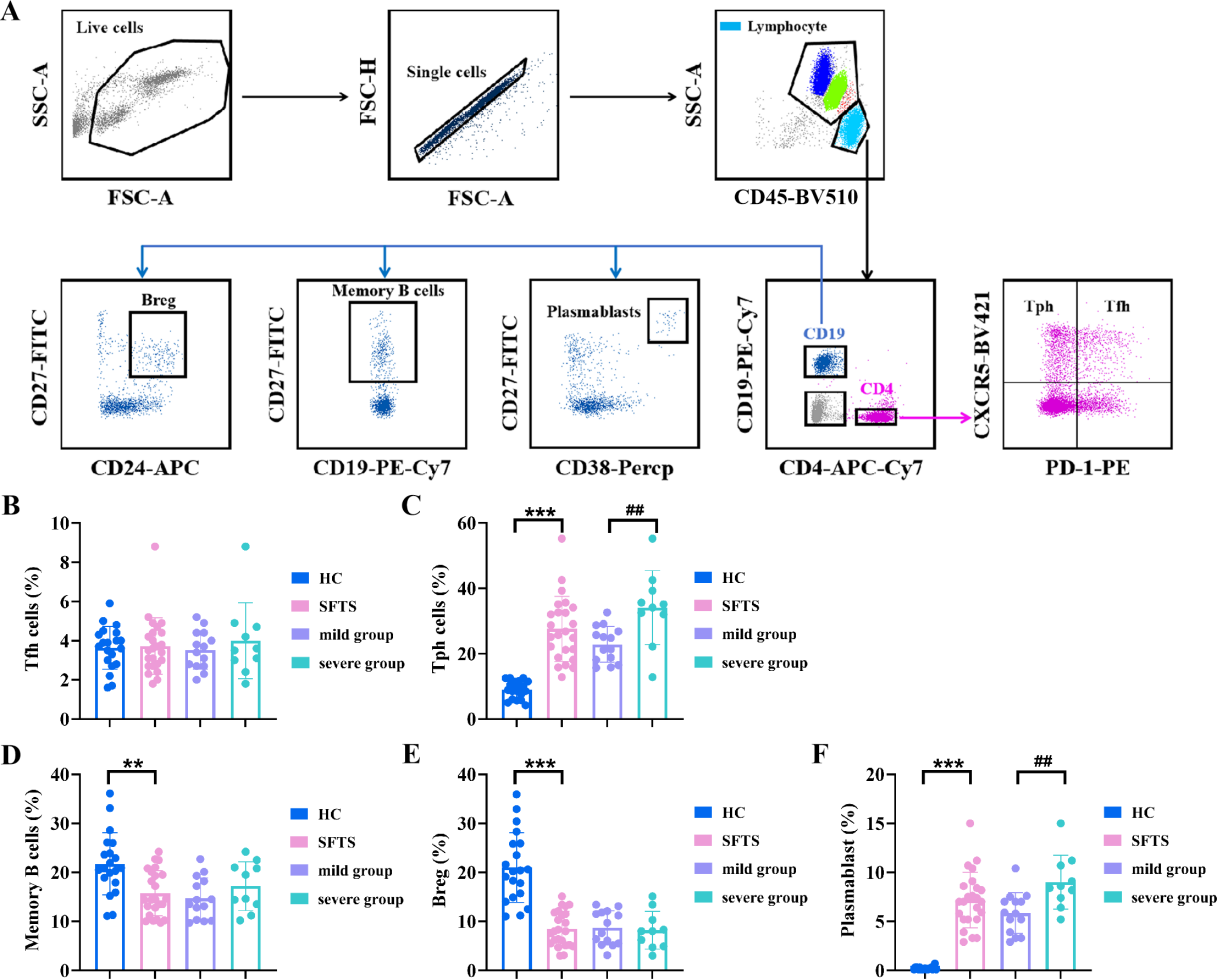
Characterization of T- and B-cell subsets in SFTS patients and HC. **(A)** Representative flow cytometry charts depicting the gating strategy for T- and B- cell subsets. **(B-F)** Analysis of the percentage of Tfh cells, Tph cells, memory B cells, Breg cells and plasmablasts in SFTS patients and HC. *Data were compared between SFTS patients and HC. #Data were compared between mild group and severe group in patients with SFTS. **p < 0.01, ***p < 0.001, ##*p* < 0.01.

Host adaptive immune responses to viruses involve T and B cells, which specifically recognize and eliminate viral pathogens. SFTSV can achieve immune escape by modulating T- and B-cell immune responses through various mechanisms. In this study, we analyzed the predominant phenotypes of T and B cells in peripheral blood of SFTS patients and HC using flow cytometry (**Figure 3A**), including T follicular helper (Tfh) cells, T peripheral helper (Tph) cells, memory B cells, regulatory B (Breg) cells, and plasmablasts. Our results revealed that SFTS patients had significantly higher frequencies of Tph cells compared to HC (**Figures 3B, C**). Besides, the percentage of plasmablasts was markedly increased in SFTS patients, while the proportions of memory B cells and Breg cells were reduced (**Figures 3D-F**). We also compared T- and B-cell subsets between the mild and severe groups in SFTS patients. As shown in **Figures 3B–F**, the frequencies of Tph cells and plasmablasts were higher in the severe group than in the mild group, whereas the population of Tfh cells, memory B cells, and Breg cells were comparable between the two groups.

### Significant expansion of peripheral plasma cells in SFTS patients

**Figure 4:**
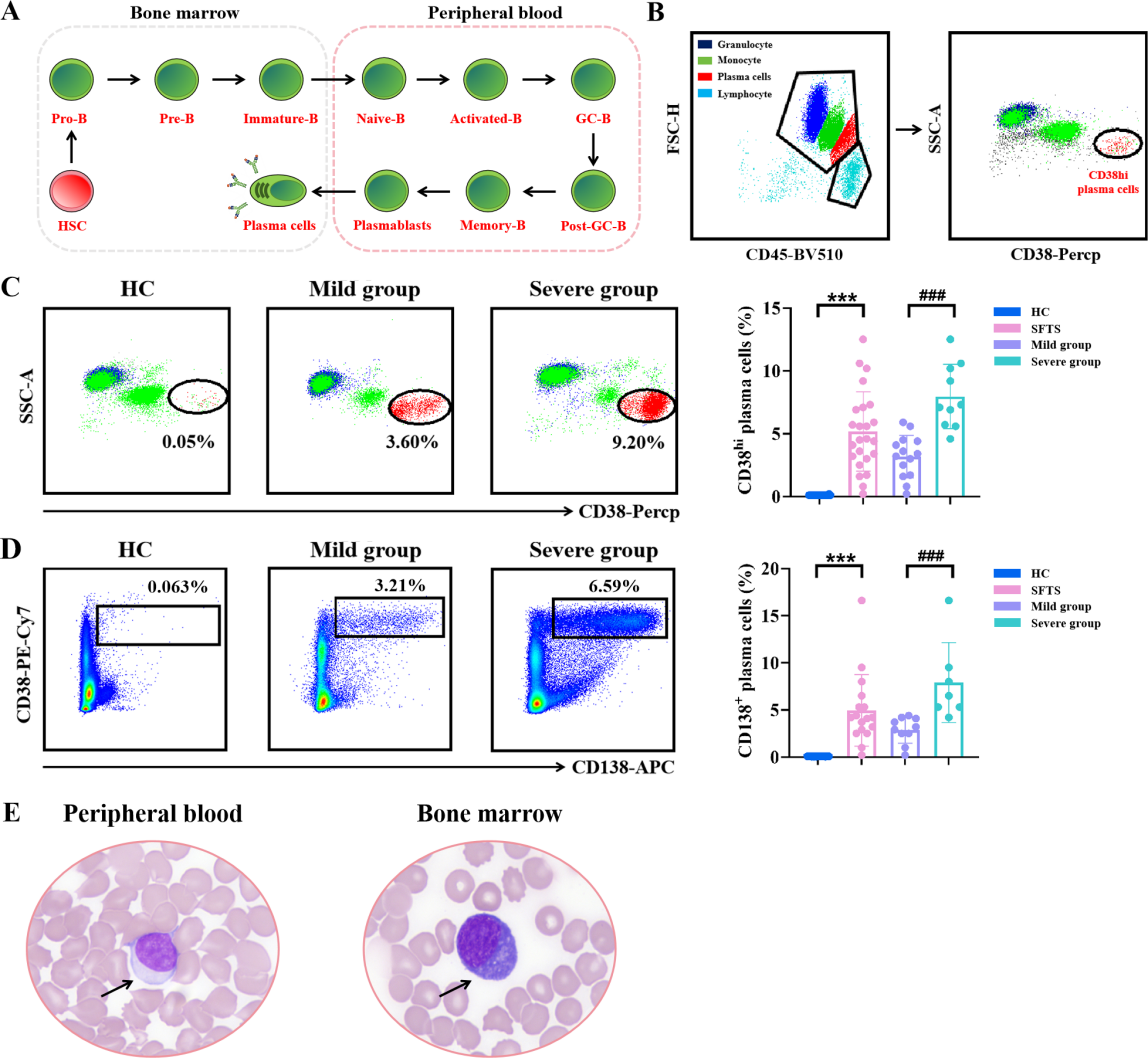
Expansion of peripheral plasma cells in SFTS patients. **(A)** Overview of the differentiation stages of B cells. **(B)** Representative flow cytometry charts depicting the gating strategy for CD38^hi^ plasma cells. **(C)** Analysis of the percentage of CD38^hi^ plasma cells in peripheral blood between SFTS patients and HC. **(D)** Analysis of the percentage of CD138^+^ plasma cells in peripheral blood between SFTS patients and HC. **(E)** Morphology of atypical lymphocytes in peripheral blood and bone marrow samples from SFTS patients. *Data were compared between SFTS patients and HC. #Data were compared between mild group and severe group in patients with SFTS. *** p < 0.001, ### p < 0.001.

At steady state, healthy humans typically have a low proportion of plasma cells in circulation. However, during acute viral infections, plasma cells can rapidly enter the bloodstream (30). In the present study, we observed a significant expansion of plasma cells in peripheral blood of SFTS patients. These plasma cells exhibited intermediate CD45 expression and higher light scatter than lymphocytes, along with elevated CD38 expression (**Figures 4A–C**). Notably, the frequency of plasma cells was significantly higher in the severe group compared to the mild group. Plasma cells are characteristically defined by the co-expression of CD138 and CD38. To identify plasma cells, we used the combination of CD38 and CD138 expression in flow cytometry analysis. As shown in **Figure 4D**, a low frequency of CD138^+^ plasma cells was detected in peripheral blood of HC, whereas significantly higher frequencies of CD138^+^ plasma cells were observed SFTS patients, constituting up to 8% of all circulating single cells. Additionally, the population of CD138^+^ plasma cells was higher in the severe group compared to the mild group (**Figure 4D**).

Due to thrombocytopenia, leukopenia, and anemia observed in some SFTS patients, bone marrow aspiration was performed to rule out hematologic disorders. In this study, bone marrow aspiration was conducted on 9 patients diagnosed with SFTS, and samples were obtained for analysis. Flow cytometry was used to assess the proportion of CD138^+^ plasma cells in bone marrow of these patients. As shown in **Supplementary Figure 1A**, the frequency of CD138^+^ plasma cells was significantly higher in SFTS patients compared to HC. Additionally, microscopic examination revealed that both peripheral blood and bone marrow samples from SFTS patients contained predominantly atypical lymphocytes. These cells were characterized by medium cell size, basophilic cytoplasm lacking cytoplasmic granules, and a wheel-like nucleus (**Figure 4E**). These morphological features further support the presence of plasma cells in peripheral blood and bone marrow of SFTSV-infected patients.

### Characteristics of peripherally expanded plasma cells in SFTS patients

**Figure 5:**
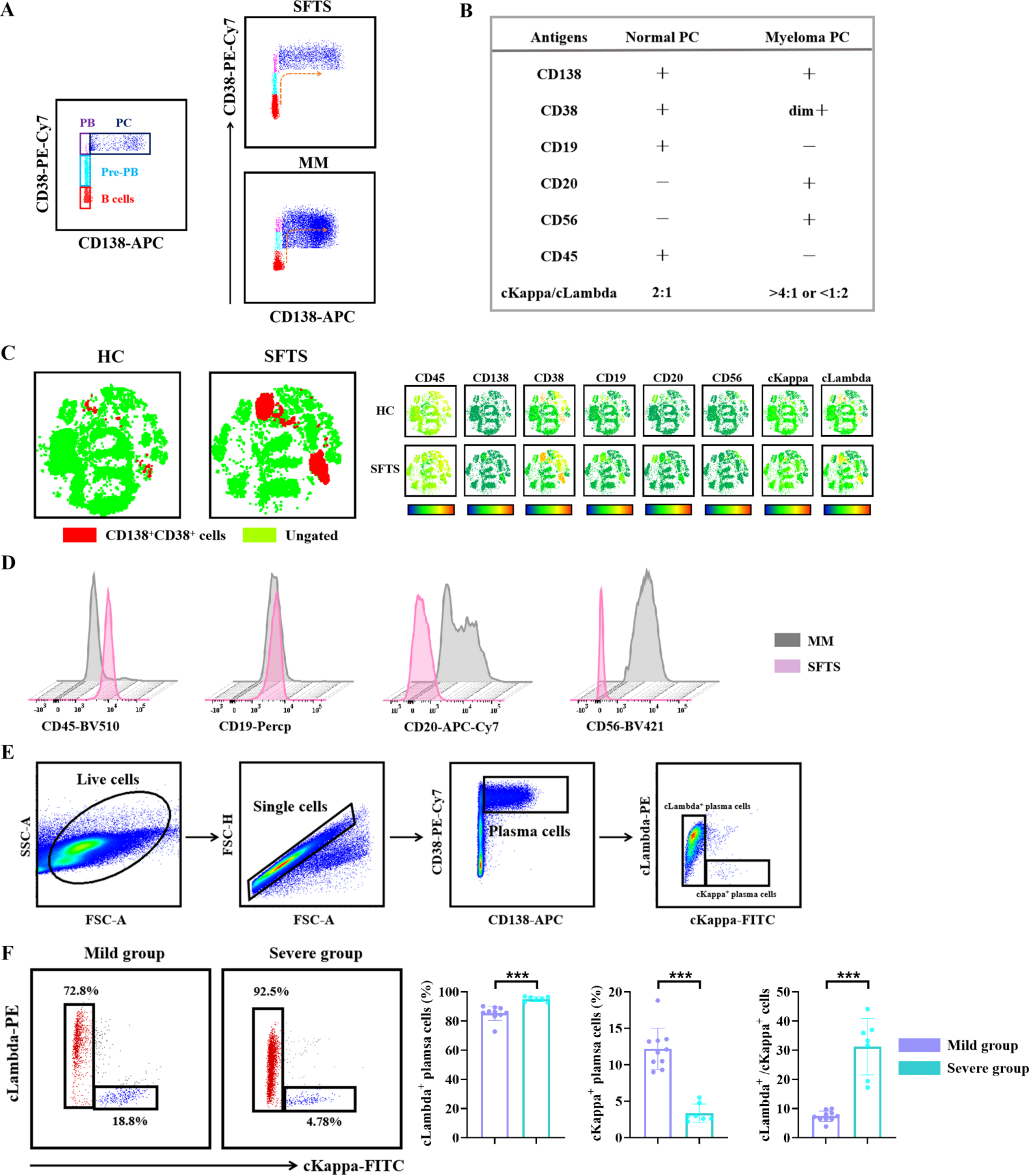
**(A)** Developmental process of B cells. Left:Developmental process of B cells in bone marrow of healthy individuals. Right: Developmental process of B cells in peripheral blood of SFTS and MM patients. **(B)** Surface molecular expression characteristics in normal versus abnormal plasma cells. **(C)** Heatmap representing scaled expression values of CD138^+^ plasma cells in peripheral blood between SFTS patients and HC. **(D)** Analysis of the expression of CD19, CD20, CD56, and CD45 in peripheral blood of SFTS and MM patients. **(E)** Representative flow cytometry charts depicting the gating strategy for CD138^+^ plasma cells. **(F)** Assessment of the cLambda^+^ plasma cells and cKappa^+^ plasma cells across different groups of SFTS patients. *** *p* < 0.001.

Multiple myeloma (MM) is a neoplastic disorder of plasma cells characterized by the clonal proliferation of plasma cells in bone marrow (31). These malignant plasma cell cells can migrate to peripheral blood, leading to in the formation of circulating plasma cells, which may transform into highly aggressive plasma cell leukemia (32). In the present study, we compared the characteristics of plasma cells in peripheral blood between SFTS and MM patients. First, we mapped the B cell developmental process, classifying the cells into B cells, pre-plasmablasts, plasmablasts, and plasma cells based on CD138 and CD38 expression (**Figure 5A**). In SFTS patients, CD38 expression varied across the sequential stages of B cell maturation. CD38 was detectable in pre-plasmablasts and progressively increased as differentiation advanced to the plasma cells stage. This finding suggests that plasma cells in SFTS patients develop normally, unlike the aberrant plasma cells development observed in MM patients.

Subsequently, to visualize the immunophenotypic characterization of plasma cells in SFTS patients, we analyzed the expression of CD19, CD20, CD56, and CD45 in peripheral blood from SFTS and MM patients. These markers are key for distinguishing benign from malignant plasma cells, as normal plasma cells typically exhibit a CD45^+^CD19^+^CD20^-^CD56^-^ phenotype. In this analysis, we revealed that the predominant immunophenotype of plasma cells in SFTS patients was characterized by the expression of CD45 and CD19, while lacking expression of CD20 and CD56, a pattern distinctly different from the malignantly expanded plasma cells found in MM patients (**Figures 5B–D**). In addition to assessing surface markers, we performed Kappa and Lambda light chains analysis to confirm the clonality of plasma cells. As shown in **Figures 5E, F**, the frequency of cLambda^+^ plasma cells was significantly increased in the severe group compared to the mild group, while the proportion of cKappa^+^ plasma cells was reduced in the severe group of SFTS patients. Moreover, the ratio of cLambda^+^ to cKappa^+^ plasma cells was notably higher in the severe group (median 33.28%, interquartile range, 25.53%-36.42%) than in the mild group (median 7.26%, interquartile range, 6.40%-8.34%), indicating that the expanded plasma cells in peripheral blood of the SFTS patients predominantly exhibited Lambda light chain expression. Furthermore, plasma cells present in bone marrow of SFTS patients exhibited characteristics consistent with those of plasma cells found in peripheral blood (**Supplementary Figures 1B–D**). Collectively, these results suggest that SFTSV infection likely induces the expansion of plasma cells with a normal immunophenotype and Lambda light chain expression.

### Peripheral expanded Lambda-expressing plasma cells are positively correlated with clinical severity in SFTS

**Figure 6:**
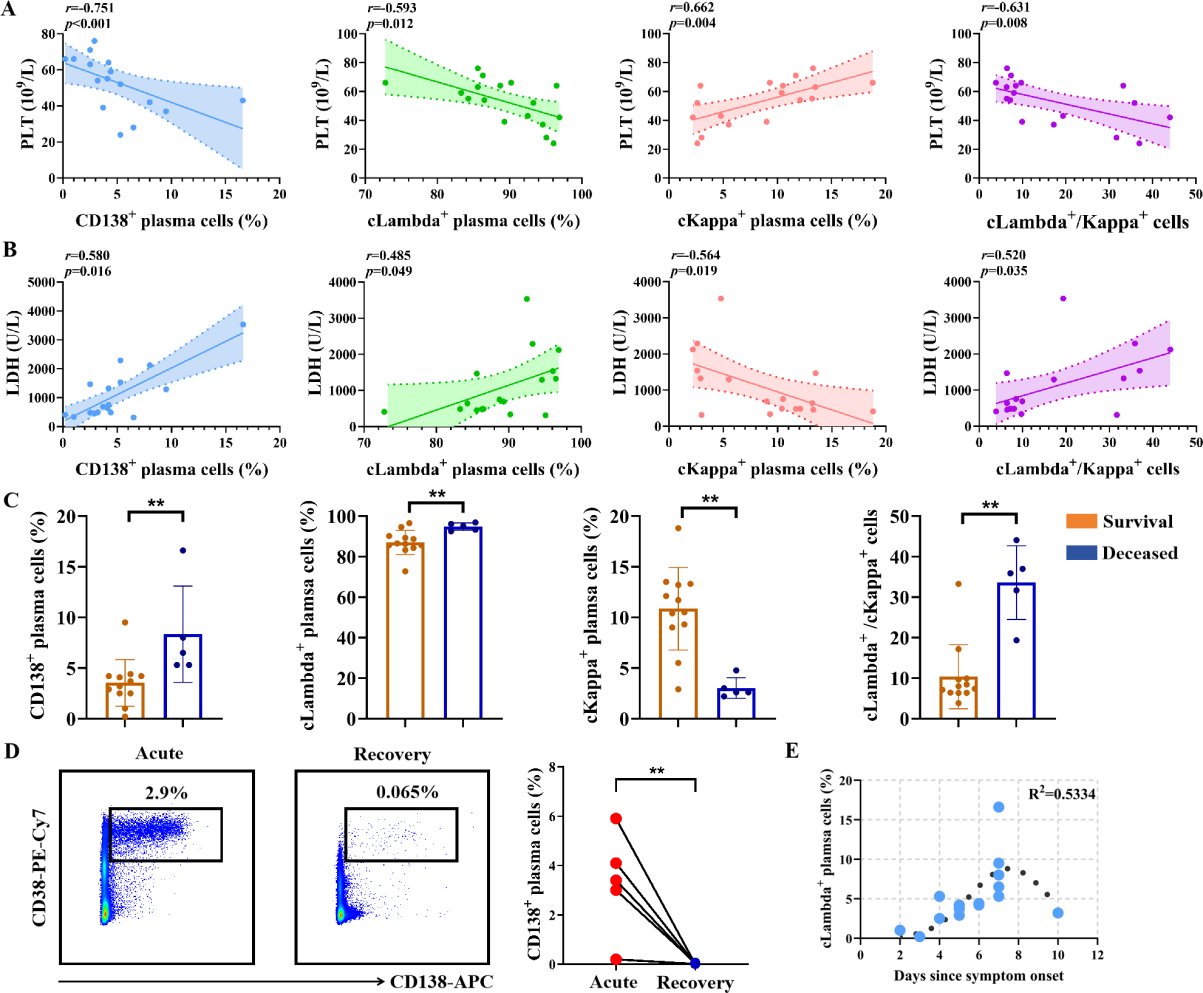
Correlation analysis between the frequency of plasma cells and clinical severity in SFTS patients. **(A, B)** Correlation between the frequencies of CD138^+^ plasma cells, cLambda^+^ plasma cells, cKappa^+^ plasma cells, and the ratio of cLambda^+^/cKappa^+^ plasma cells with PLT count (A) and LDH level **(B)**. **(C)** Analysis of the percentages of CD138^+^ plasma cells, cLambda^+^ plasma cells, cKappa^+^ plasma cells, and the ratio of cLambda^+^/cKappa^+^ plasma cells in peripheral blood samples between the survival and the deceased group of SFTS patients. **(D)** Comparison of the frequency of CD138^+^ plasma cells between the acute and recovery phases of SFTSV infection. **(E)** Kinetics of cLambda^+^ plasma cells frequency in peripheral blood from SFTS patients. ** *p* < 0.01.

To investigate the relationship between plasma cells in peripheral blood and the clinical outcomes of SFTSV-infected patients, we performed a correlation analysis between plasma cell subsets and laboratory characteristics. As shown in **Figures 6A, B**, the populations of plasma cells, cLambda^+^ plasma cells, and the ratio of cLambda^+^/cKappa^+^ plasma cells exhibited a negative correlation with PTL count, while showing a positive correlation with LDH level. In contrast, the frequency of cKappa^+^ plasma cells was positively correlated with PTL count and negatively correlated with LDH level. Besides, we observed a significant positive correlation between the frequency of circulating cLambda^+^ plasma cells and serum IL-6 level. Detailed information on the correlation analysis between plasma cell subsets and other clinical characteristics of SFTS patients is provided in **Supplementary Table 2**.

We further compared the differences in plasma cell subsets in peripheral blood between the survival group (n=12) and the deceased group (n=5) of SFTS patients. As shown in **Figure 6C**, the frequencies of plasma cells, cLambda^+^ plasma cells, and the ratio of cLambda^+^/cKappa^+^ plasma cells were significantly higher in the survival group compared to the deceased group. In contrast, the proportion of cKappa^+^ plasma cells was lower in the deceased group. Additionally, we evaluated the changes in plasma cell subsets in peripheral blood before and after effective therapy for SFTS patients. As shown in **Figure 6D**, the median frequency of plasma cells was 2.9% (interquartile range, 2.5%-4.1%) during the acute phase, which significantly decreased to 0.013% (interquartile range, 0.012%-0.022%) in the recovery phase. Besides, we observed that the frequency of cLambda^+^ plasma cells gradually increased following symptom onset, peaked at day 7, and subsequently declined (**Figure 6E**). Taken together, these findings suggest that the number of plasma cells in peripheral blood returned to near-normal levels after recovery from SFTSV infection, and the transient expansion of Lambda-expressing plasma cells in peripheral blood was notably distinct from that seen in MM. 

### Peripheral expanded Lambda-expressing plasma cells are a distinctive immunological hallmark of SFTSV infection

**Figure 7:**
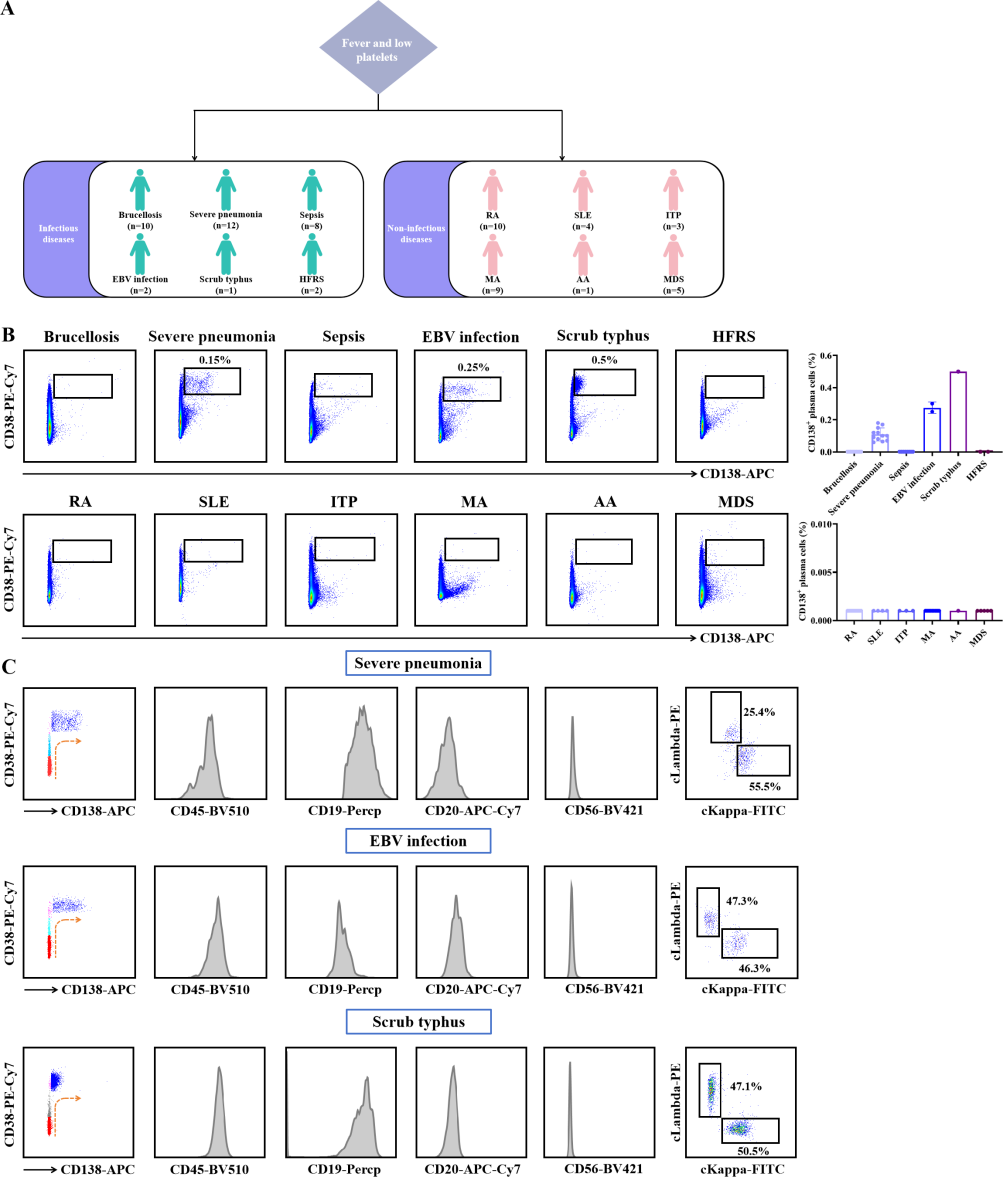
Characterization of peripheral plasma cells in infectious and non-infectious diseases. **(A)** Flow chart of patients with infectious diseases and non-infectious diseases enrolled in the study. **(B)** Analysis of the population of CD138^+^ plasma cells in peripheral blood. **(C)** Immunophenotypic characteristics of CD138^+^ plasma cells in peripheral blood of patients with severe pneumonia, EBV infection, and scrub typhus.

Given the marked increase in the population of peripheral plasma cells and the distinct immunophenotypic characteristics identified in SFTS patients, we performed a comparative analysis of plasma cells alterations in SFTS and other clinically similar diseases, including infectious diseases and non-infectious conditions (**Figure 7A**). As shown in **Figure 7B**, plasma cells were detected in peripheral blood of patients with severe pneumonia, EBV infection, and scrub typhus. However, no expansion of plasma cells was observed in patients with brucellosis, sepsis, HFRS, RA, SLE, ITP, MA, AA, or MDS. Further analysis was conducted to characterize plasma cells in cases of severe pneumonia, EBV infection, and scrub typhus. While no significant differences were observed in the development process or surface molecule expression of plasma cells between patients with SFTS and those with severe pneumonia, EBV infection, and scrub typhus. However, the ratio of cLambda^+^ to cKappa^+^ plasma cells remained within the normal range in these patients, in contrast to the findings observed in SFTS patients (**Figure 7C**). Based on the unique immunophenotypic characteristics of peripheral blood plasma cells in SFTS patients, we developed a flowchart to streamline the rapid screening of SFTS patients through flow cytometry analysis (**Supplementary Figure 2**). In patients presenting with fever and thrombocytopenia, the detection of an elevated number of plasma cells in peripheral blood, along with a normal development process, immunophenotype, and positive Lambda light chain expression-raises a strong suspicion of SFTSV infection. In such cases, further diagnostic measures should be pursued to confirm SFTSV infection, while ruling out other potential diagnoses if these criteria are not met.

### Validation of Lambda-expressing plasma cells expansion in and independent cohort

**Figure 8:**
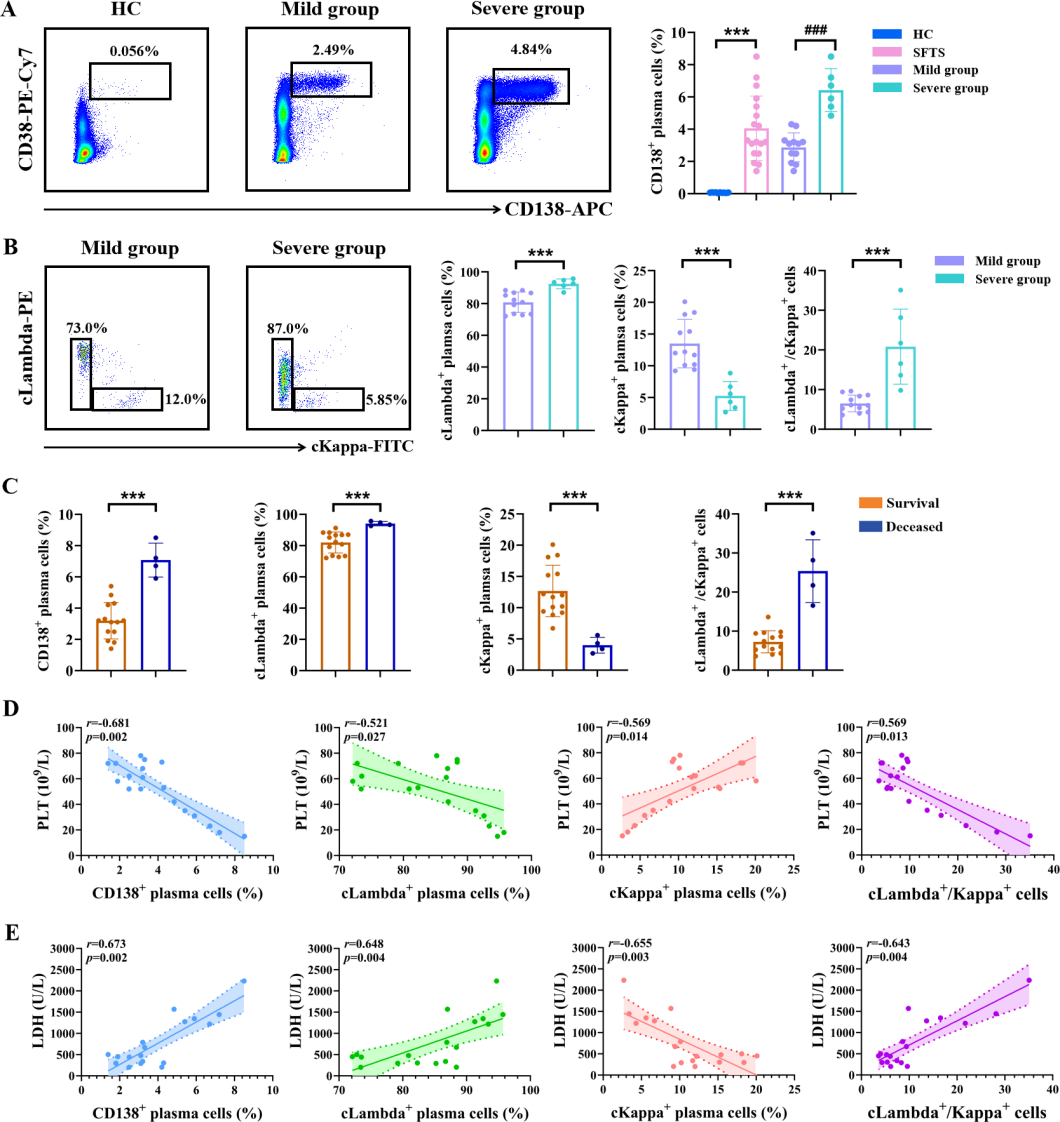
Validation of Lambda-expressing plasma cells expansion in and independent cohort. **(A)** Analysis of the percentage of CD138^+^ plasma cells in peripheral blood between SFTS patients and HC. **(B)** Assessment of the cLambda^+^ plasma cells and cKappa^+^ plasma cells across different groups of SFTS patients. **(C)** Compared the frequencies of plasma cell subsets between the survival and the deceased group of SFTS patients. **(D, E)** Correlation between the frequencies of CD138^+^ plasma cells, cLambda^+^ plasma cells, cKappa^+^ plasma cells, and the ratio of cLambda^+^/cKappa^+^ plasma cells with PLT count **(D)** and LDH level **(E)**. *** *p* < 0.001, ### *p* < 0.001.

To validate Lambda-expressing plasma cells expansion in patients with SFTS, we established an independent validation cohort comprising 18 patients with SFTS and 10 HC admitted to the Dalian Public Health Clinical Center between October 2024 and October 2025. The demographic and clinical characteristics of the validation cohort are summarized in **Supplementary Table 3**. As shown in **Figure 8A**, the frequency of CD138^+^ plasma cells was markedly higher in SFTS patients than in HC. Notably, the proportion of cLambda^+^ plasma cells was significantly increased in the severe group compared to the mild group (**Figure 8B**). We further compared the frequency of cLambda^+^ plasma cells between survival (n=14) and deceased (n=4) SFTS patients. As shown in **Figure 8C**, the number of cLambda^+^ plasma cells was significantly higher in the survival group than in the deceased group. Correlation analysis revealed that the population of cLambda^+^ plasma cells was negatively correlation with PTL count and positively correlation with LDH level (**Figures 8D, E**). Consequently, these findings from an independent external cohort confirm that the expansion of Lambda-expressing plasma cells is a characteristic feature of SFTS and is closely associated with disease severity and prognosis.

## Discussion

Defining the specific immune responses to SFTSV infection in humans is essential for identifying a protective profile that can aid in pathogenesis studies and vaccine development. In this study, we demonstrated a highly activated and exhausted T cell phenotype in SFTS patients, along with a significant expansion of Tph cells and plasmablasts. Notably, the transient expansion of Lambda-expressing plasma cells in circulation could serve as a distinctive immunological hallmark of SFTSV infection, with levels correlating with disease severity and prognosis. Moreover, SFTS can be differentiated from other febrile or thrombocytopenic conditions based on the immunophenotypic characteristics of peripheral plasma cells.

T lymphocytes are key immune cells that mediate cellular immune responses, with their role in regulating viral infections (33). Upon viral infection, naïve T cells become activated, proliferate, and differentiate into cytotoxic and effector T cells, which play a critical role in pathogen clearance. Previous reports have shown the numbers of CD4^+^ and CD8^+^ T cells are significantly reduced in both surviving and deceased SFTS patients during the acute phase (10). Additionally, the activation and exhaustion of T cells are associated with the worsening of SFTS (11). Our findings align with these previous studies, emphasizing the importance of T cell activation and exhaustion in SFTS. Furthermore, we observed that patients with severe infection exhibited a marked increase in the activation and exhaustion of CD4^+^ T cells compared to patients with mild infection, whereas no significant differences in CD8^+^ T cell activation or exhaustion were noted between these groups. This suggests that the activation and exhaustion of CD4^+^ T cells, rather than CD8^+^ T cells, play a critical role in the severity of SFTS. Notably, anti-PD-1 immunotherapy has proven effective and safe in managing persistent HCV infection in chimpanzees (34), highlighting PD-1 as a potential therapeutic target for SFTSV infection.

T-B cell interaction is crucial for protecting the host from severe viral infections. It is well-established that Tfh and Tph cells are the primary T cell subsets that exhibit “B cell help” signatures and induce B cell differentiation and maturation (35). Unlike Tfh cells, which interact with B cells in lymphoid organs, Tph cells provide assistance to B cells in inflamed tissues (36). In this study, we examined the changes in Tfh and Tph cells during SFTSV infection and found that SFTS patients exhibited a significantly elevated the frequency of Tph cells, while the population of Tfh cells showed no significant difference between the two groups. Moreover, the percentage of Tph cells was notably higher in the severe group as compared to in the mild group of SFTS patients. These findings suggest that the expansion of Tph cells is critical for the humoral response to SFTSV infection and for the regulation of viral pathogenesis. Additionally, Song et al. (12) observed an expansion of Tfh cells during the early stages of SFTSV infection, noting that the number of Tfh cells correlated with the recovery status of the disease. The discrepancy in results regarding Tfh cells may be due to differences in the definition of Tfh cells, disease severity, and sample size across these studies.

B cells are suspected to be the primary targets of SFTSV infection, and the impairment of humoral immunity has been previously reported in SFTS patients (13). Postmortem analyses of lymph nodes revealed that activated mature B cells of the plasmablast lineage were abundant in the secondary lymphoid organs of deceased SFTS cases (14). Additionally, IFN-I-inducible plasmablasts are susceptible to SFTSV infection and may serve as an important reservoir in fatal cases (7). Several studies have shown that the absence of a virus-specific IgG response is associated with fatal outcomes in SFTS patients (12, 37). In this study, we observed a disruption in the balance of B cell subsets, characterized by an increase in plasmablasts and a decrease in memory B cells and Breg cells. Moreover, a higher proportion of plasmablasts was detected in patients with severe compared to those with mild cases. These results suggest that a large number of naïve B cells were primed into plasmablasts, and the overproliferation of plasmablasts is a key feature of SFTS. More intriguingly, our analysis revealed a positive correlation between the frequency of Tph cells and plasmablasts in the peripheral blood of patients with SFTS (data not shown). It is strongly postulated that Tph cells play a pivotal role in the rapid expansion of plasmablasts during the acute phase of SFTSV infection. These findings highlight how T cells can drive B cell differentiation in the context of SFTSV infection and provide insights into the role of Tph cells in mediating aberrant T-B cell interactions in SFTS. A recent study reported that Tph cells were significantly increased and positively correlated with the frequency of plasmablasts in the blood of patients with COVID-19 (38), which is partly consistent with our results. Breg cells, which express IL-10, are recognized as an important new subset of B cells that help restrain excessive inflammatory responses during infection, inflammation, and autoimmune diseases (39, 40). Importantly, the impairment of Breg cells contributes to immune dysfunction in HIV and HBV infections. In our study, the frequency of Breg cells was significantly reduced in SFTS patients, suggesting that the imbalance between effector and regulatory B cells may contribute to the pathogenesis of SFTS. Therefore, future strategies to treat SFTS may focus on increasing the number of Breg cells or reducing the number of effector B cells.

Based on flow cytometry analysis of immune cell subsets, we identified a significant expansion of plasma cells in peripheral blood of patients with SFTS during the acute phase. Additionally, morphological characteristics further corroborate the presence of plasma cells in SFTSV-infected patients. Notably, the excessive proliferation of plasma cells exhibited a transient presence in peripheral blood, with the number of plasma cells returning to near-normal levels once the SFTS patients recovered. Plasma cells play a crucial role in producing antibodies that neutralize the viruses during the human immune response to viral infections. Under steady-state conditions, healthy humans have a low proportion of plasma cells in circulation, but during acute viral infections, plasma cells can rapidly enter the bloodstream. Previous studies have reported the presence of transient plasma cells in infections caused by hantavirus, Hepatitis A virus, dengue virus, and Ebola virus (41–45). Regarding SFTSV infection, several case reports have described atypical lymphocytes in peripheral blood and bone marrow of patients with SFTS (46, 47). A recent study involving 30 SFTS cases suggested that the overproliferation of plasma cells in bone marrow is positively correlated with clinical severity (48). Moreover, Park et al. (37) observed that plasma cells were the primary cell type harboring SFTSV RNAs and suggested that SFTSV-infected B cells exhibited reduced IFN signaling compared to uninfected B cells. Here, we present the phenomenon of reactive plasma cells characterized by a transient increase in peripheral blood as being particularly associated with SFTSV infection. In cases of SFTS, plasma cells can account for up to 8% of all circulating single cells, and their frequency is positively correlated with clinical severity. Notably, our study highlights the presence of both plasmablasts and plasma cells in peripheral blood during SFTSV infection. It is important to differentiate between these two cell types in the analysis. A study by Huang et al. (10) reported a significant increase in the proportion of plasmablasts (CD27^hi^CD38^hi^) within the total B cell population in SFTS patients compared to HC. In their study, the identification of plasmablasts within the lymphocyte population was based on CD38 expression, and these cells constituted approximately 50% of the total B cell population-markedly higher than the proportion observed in our study. We speculate that this difference may be due to the ignorance of plasma cells in peripheral blood analysis of SFTSV-infected patients.

Overproliferation of plasma cells is commonly observed in plasma cell neoplasms, such as MM, Waldenström’s macroglobulinemia, and systemic amyloidosis (49). In MM, plasma cell clones become dysregulated, continuously proliferating and secreting immunoglobulins, which can be identified as a monoclonal immunoglobulin peak in the patient’s serum (50). It is crucial to differentiate between normal and malignant plasma cells based on their immunophenotype when plasma cells expansion is observed in peripheral blood. In this study, we showed that the plasma cells observed in patients with SFTS were B-lineage lymphocytes with normal differentiation into plasma cells, characterized by the expression of CD45 and CD19 while lacking CD20 and CD56 expression. This immunophenotype was distinctly different from the phenotype of malignantly expanded plasma cells found in patients with MM. In addition to surface marker analysis, we performed Kappa and Lambda light chain analysis to confirm the clonality of plasma cells in SFTSV-infected patients. Surprisingly, overproliferation of circulating Lambda-type plasma cell subsets was identified in SFTS cases, similar to the pattern seen in Lambda light chain MM. A serum immunofixation test further revealed a small amount of Lambda chain-type monoclonal protein (data not shown). Furthermore, Lambda-expressing plasma cells were also observed in bone marrow of SFTS patients. To our knowledge, this transient expansion of Lambda-expressing plasma cells induced by SFTSV infection has not been systematically reported in peripheral blood. In 2018, Zhang et al. (46) described an SFTS case with abnormal monoclonal Lambda-expressing plasma cells in bone marrow, which aligns with our findings. Recent reports have also noted the presence of Lambda-type monoclonal plasma cells in bone marrow of SFTSV-infected patients (48). Notably, the overproliferation of Lambda-expressing plasma cells in SFTSV infection is distinctly from Lambda light chain MM, as these cells in SFTS patients appear to be transient. Therefore, immunophenotypic and clonality assessments of plasma cells are essential to avoid misdiagnosis of SFTSV infection as Lambda light chain MM or other plasma cell neoplasms. It is important to note that while we have confirmed Lambda light chain restriction in plasma cells from SFTS patients via flow cytometric light chain analysis, definitive validation of the clonal origin of these plasma cells through NGS-based immunoglobulin gene rearrangement analysis remains to be completed in future studies.

Importantly, the mechanism underlying the observed phenomena in SFTS cases remains unclear. Several studies have suggested that SFTSV infection can trigger a cytokine storm, with elevated levels of serum cytokines such as IL-6, IL-10, MCP-1, G-CSF, and IFN-γ, which may contribute to disease severity and outcomes (51–52). Among these, IL-6 is involved in the differentiation of B cells into plasma cells and influences immunoglobulin secretion in plasma cells as demonstrated in knockout mice (53). Additionally, IL-6 plays a central role in the development of malignant plasma cells, and the injection of anti-IL-6 monoclonal antibodies has been shown to inhibit myeloma cell proliferation and reduce the number of myeloma cells by 50% in cultures derived from patients' bone marrow cells *in vitro (*54, 55). In this study, we observed a significant positive correlation between serum IL-6 level and the frequency of circulating cLambdaValidation of Lambda^+^ plasma cells. Based on these findings, we hypothesize that excessive IL-6 production could be a potential explanation for the expansion of Lambda-expressing plasma cells in SFTSV infection. Further studies are needed to explore the role of IL-6 in driving cLambda^+^ plasma cells expansion in SFTS.

SFTS generally presents with an acute onset, characterized by clinical symptoms such as high fever, fatigue, myalgia, chills, headache, lymphadenopathy, gastrointestinal disturbances, and a hemorrhagic tendency. Due to the nonspecific nature of these symptoms, which can involve multiple organ systems, many SFTS patients are misdiagnosed with other diseases such as common fever, gastrointestinal disorders, human granulocytic anaplasmosis, HFRS, or leptospirosis (56). Reports suggest that 30-80% of suspected SFTS cases test positive for SFTSV, while others are misdiagnosed (57). Early diagnosis and prompt treatment are essential for improving patient survival and prognosis. In the present study, we performed a comparative analysis of plasma cell alterations in SFTS and several clinically similar diseases, revealing the presence of plasma cells in peripheral blood of patients with severe pneumonia, EBV infection, and scrub typhus. Further characterization of these plasma cells showed that their development and surface marker expression were similar to those observed in SFTS patients. Notably, the ratio of cLambda^+^ to cKappa^+^ plasma cells remained within the normal range in these patients, unlike the findings in SFTSV infection. These findings suggest that the peripheral expansion of Lambda-expressing plasma cells is a distinctive immunological hallmark of SFTSV infection and support the potential for early screening based on plasma cells immunophenotype. Based on the unique immunophenotypic characteristics of peripheral blood plasma cells in SFTS patients, we developed a flowchart to streamline the rapid screening of SFTS patients through flow cytometry analysis. This finding provides a valuable reference for the early differential diagnosis of SFTS, facilitates the rapid clinical diagnosis of the disease, and reduces its case fatality rate.

Our findings have significant implications for clinicians to some extent. Bone marrow examination has traditionally been used to rule out hematologic disorders in SFTS studies. However, bone marrow aspiration is a painful procedure, and frequent sampling for disease monitoring presents a clinical challenge. Based on the results of our study, the transient expansion of Lambda-expressing plasma cells observed in both peripheral blood and bone marrow, may serve as a distinctive immunological hallmark of SFTSV infection. Clinicians should be aware that bone marrow aspiration is not necessary for diagnosing SFTS. Instead, peripheral blood examination using flow cytometry analysis could provide valuable insights, avoiding the need for bone marrow sampling and aiding in clinical diagnosis. Additionally, evaluating the immunophenotype and clonality of plasma cells in SFTSV-infected patients is crucial, as this assessment can help prevent misdiagnosis and delays in diagnosis.

This study provides valuable insights, but there are several limitations that must be acknowledged. First, the sample size was small, and thus larger-scale studies are required. Second, while we identified a particular phenomenon associated with SFTSV infection-the transient expansion of Lambda-expressing plasma cells, the underlying mechanism and potential role of these cells in SFTS remain unclear. Further research is needed to address these questions. Third, although we performed a comparative analysis of plasma cells alterations in SFTS and several clinically similar diseases was performed, some diseases that are easily misdiagnosed as SFTS, such as human granulocytic anaplasmosis, leptospirosis, dengue fever, and typhoid fever, were not included in the study. In subsequent studies, we will establish multi-center collaborations to recruit larger and more heterogeneous cohorts of SFTS patients, and collect cases of diseases prone to misdiagnosis with SFTS to verify the accuracy and specificity of the findings in this study across different populations and clinical settings. 

In summary, we report that SFTSV infection leads to the transient expansion of Lambda-expressing plasma cells in peripheral blood, which is associated with clinical severity and patient outcomes. This study highlights a unique immunophenotype of SFTSV infection, providing important insights into the pathogenesis, prognosis, and the rational development of therapeutic strategies for SFTSV.

## Data Availability

The raw data supporting the conclusions of this article will be made available by the authors, without undue reservation.

## Supplementary material

Supplementary Figure 1 Characteristics of plasma cells in bone marrow of patients with SFTS. **(A)** Percentage of CD138^+^ plasma cells in bone marrow samples from SFTS and MM patients. **(B)** Developmental process of B cells in bone marrow of SFTS and MM patients. **(C)** The expression of CD19, CD20, CD56, and CD45 in bone marrow of SFTS and MM patients. D. The percentages of cLambda^+^ and cKappa^+^ plasma cells in different groups of SFTS patients. * *p* < 0.05.

Supplementary Figure 2 Flowchart for early diagnosis of SFTSV infection based on the characteristics of peripheral plasma cells identified through flow cytometry analysis.
