# Supplementary figures and images for: Transient expansion of peripheral Lambda-expressing plasma cells represents a distinctive phenotype associated with SFTSV infection
Source: Front Immunol. 2026 Apr 24;17:1763231. doi: 10.3389/fimmu.2026.1763231 (PMC13154158; doi:10.3389/fimmu.2026.1763231)

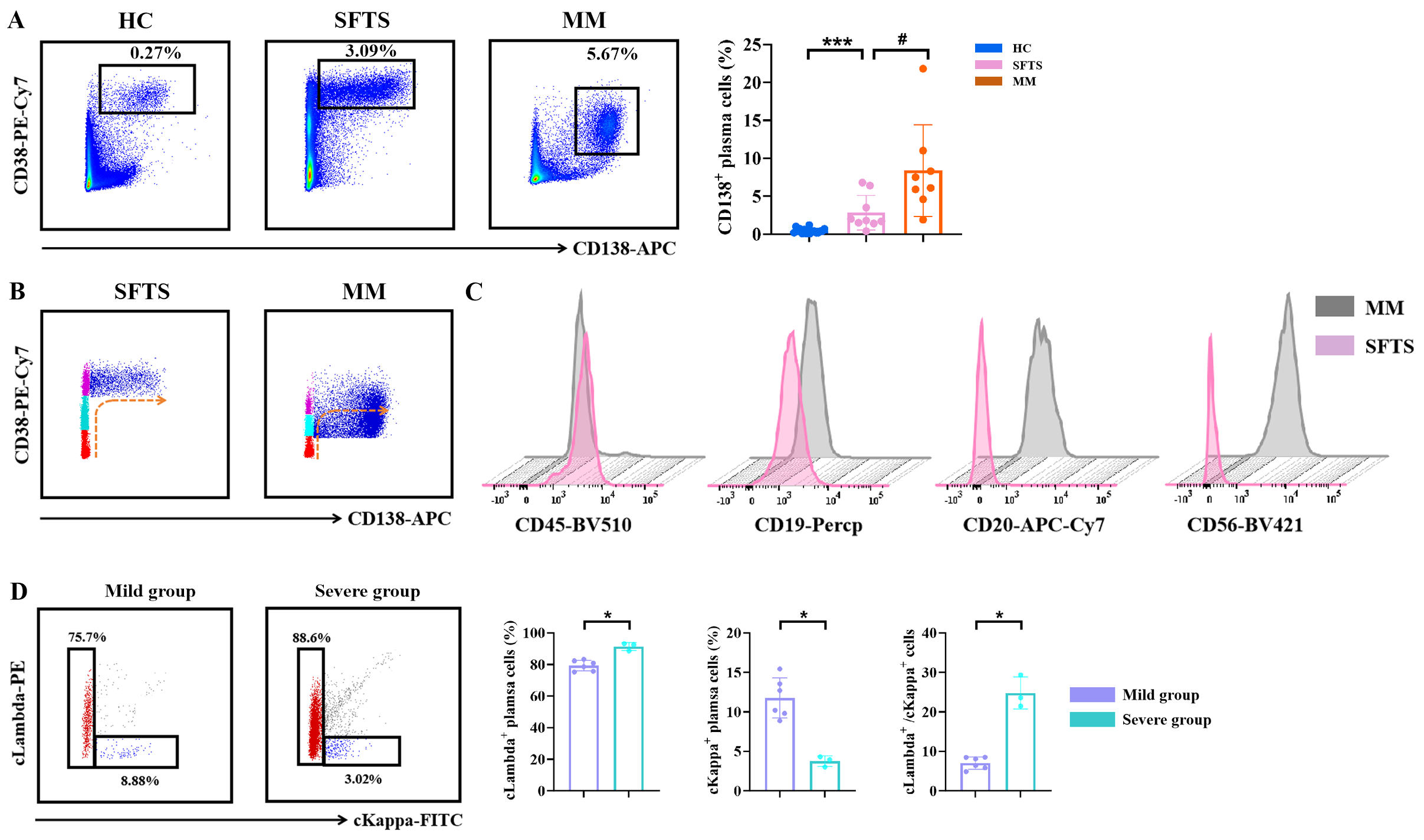

Supplement: Supplementary Figure 1 — Characteristics of plasma cells in bone marrow of patients with SFTS. (A) Percentage of CD138+ plasma cells in bone marrow samples from SFTS and MM patients. (B) Developmental process of B cells in bone marrow of SFTS and MM patients. (C) The expression of CD19, CD20, CD56, and CD45 in bone marrow of SFTS and MM patients. D. The percentages of cLambda+ and cKappa+ plasma cells in different groups of SFTS patients. * p < 0.05. [file Image1.tif]

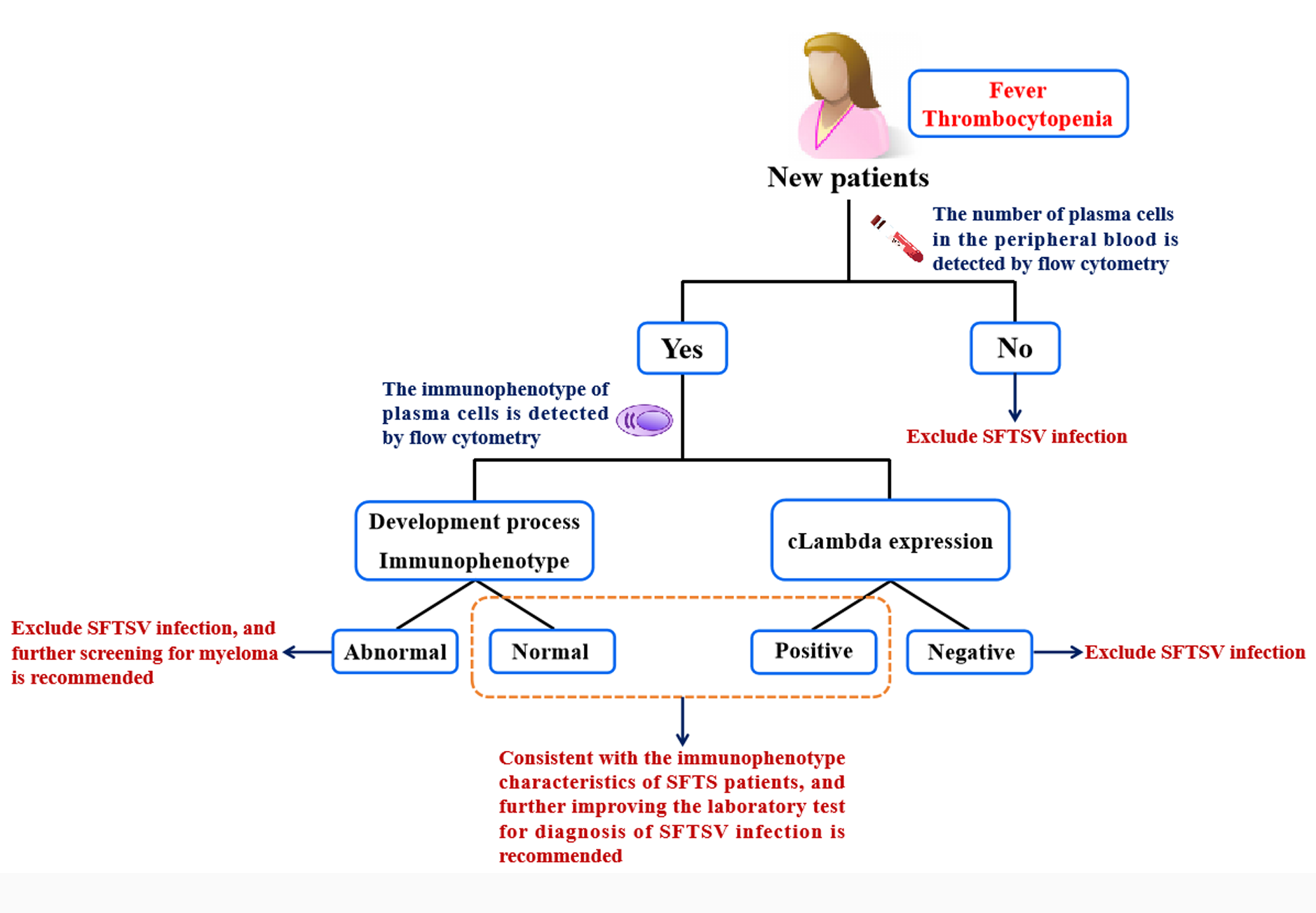

Supplement: Supplementary Figure 2 — Flowchart for early diagnosis of SFTSV infection based on the characteristics of peripheral plasma cells identified through flow cytometry analysis. [file Image2.tif]
