# Supplementary material for: Transient expansion of peripheral Lambda-expressing plasma cells represents a distinctive phenotype associated with SFTSV infection
Source: Front Immunol. 2026 Apr 24;17:1763231. doi: 10.3389/fimmu.2026.1763231 (PMC13154158; doi:10.3389/fimmu.2026.1763231)
Supplement: Supplementary file 5 [file Table1.docx]

**T****able S1. Key antibodies resources table.**

| **Antibodies** | **Source** | **Identifier** |
| --- | --- | --- |
| FITC anti-human CD38 antibody | BD | Cat# 340927 |
| PE anti-human PD-1 antibody | BD | Cat# 557946 |
| PerCP/Cyanine5.5 anti-human CD3 antibody | BD | Cat# 340949 |
| APC anti-human HLA-DR antibody | BD | Cat# 340549 |
| APC/Cyanine7 anti-human CD4 antibody | BD | Cat# 341095 |
| PE/Cyanine7 anti-human CD8 antibody | BD | Cat# 335787 |
| Brilliant Violet 421 anti-human CD56 antibody | BioLegend | Cat# 362552 |
| Brilliant Violet 510 anti-human CD45 antibody | BD | Cat# 563204 |
| FITC anti-human CD27 antibody | BioLegend | Cat# 356404 |
| PerCP/Cyanine5.5 anti-human CD38 antibody | BioLegend | Cat# 356614 |
| APC anti-human CD24 antibody | BioLegend | Cat# 382604 |
| PE/Cyanine7 anti-human CD19 antibody | BD | Cat# 341093 |
| Brilliant Violet 421 anti-human CXCR5 antibody | BioLegend | Cat# 256920 |
| FITC anti-human Kappa antibody | BD | Cat# 643773 |
| PE anti-human Lambda antibody | BD | Cat# 642919 |
| PerCP/Cyanine5.5 anti-human CD19 antibody | BioLegend | Cat# 363016 |
| APC anti-human CD138 antibody | BD | Cat# 347193 |
| APC/Cyanine7 anti-human CD20 antibody | BD | Cat# 225812 |
| PE/Cyanine7 anti-human CD38 antibody | BD | Cat# 335808 |
