# Supplementary material for: Transient expansion of peripheral Lambda-expressing plasma cells represents a distinctive phenotype associated with SFTSV infection
Source: Front Immunol. 2026 Apr 24;17:1763231. doi: 10.3389/fimmu.2026.1763231 (PMC13154158; doi:10.3389/fimmu.2026.1763231)
Supplement: Supplementary file 6 [file Table2.docx]

| **Characteristics** | **CD138^+^ plasma cells%** | | **cLambda^+^ plasma cells%** | | **cKappa^+^ plasma cells%** | | **cLambda^+^/cKappa^+^ cells** | |
| --- | --- | --- | --- | --- | --- | --- | --- | --- |
|  | ***r*** | ***p*** | ***r*** | ***p*** | ***r*** | ***p*** | ***r*** | ***p*** |
| WBC | -0.318 | 0.2112 | -0.143 | 0.585 | 0.225 | 0.385 | -0.212 | 0.414 |
| RBC | 0.047 | 0.856 | 0.228 | 0.378 | -0.268 | 0.299 | 0.248 | 0.335 |
| Monocyte | -0.160 | 0.537 | 0.033 | 0.901 | 0.087 | 0.739 | 0.007 | 0.979 |
| Lymphocyte | -0.280 | 0.275 | -0.118 | 0.652 | 0.258 | 0.317 | -0.204 | 0.430 |
| PLT | -0.751 | <0.001 | -0.593 | 0.012 | 0.662 | 0.004 | -0.631 | 0.008 |
| HGB | -0.154 | 0.553 | -0.008 | 0.976 | -0.084 | 0.748 | 0.060 | 0.818 |
| AST | 0.069 | 0.793 | 0.479 | 0.052 | -0.508 | 0.037 | 0.378 | 0.136 |
| ALT | 0.276 | 0.281 | 0.309 | 0.227 | -0.360 | 0.155 | 0.351 | 0.168 |
| ALB | -0.323 | 0.204 | -0.172 | 0.510 | 0.225 | 0.386 | -0.135 | 0.603 |
| LDH | 0.580 | 0.016 | 0.485 | 0.049 | -0.564 | 0.019 | 0.520 | 0.035 |
| cTnI | 0.2812 | 0.271 | -0.024 | 0.927 | 0.057 | 0.827 | 0.238 | 0.355 |
| CK-MB | 0.244 | 0.342 | 0.190 | 0.464 | -0.223 | 0.389 | 0.269 | 0.296 |
| APTT | 0.761 | <0.001 | 0.383 | 0.130 | -0.516 | 0.034 | 0.601 | 0.012 |
| PT | 0.242 | 0.347 | -0.172 | 0.509 | -0.046 | 0.860 | -0.020 | 0.942 |
| TT | 0.652 | 0.005 | 0.407 | 0.105 | -0.473 | 0.055 | 0.630 | 0.008 |
| D-dimer | 0.733 | 0.001 | 0.425 | 0.089 | -0.565 | 0.018 | 0.702 | 0.002 |
| Fibrinogen | 0.112 | 0.668 | 0.285 | 0.268 | -0.292 | 0.255 | -0.066 | 0.802 |
| CRP | 0.480 | 0.053 | 0.340 | 0.181 | -0.362 | 0.153 | 0.304 | 0.235 |
| PCT | 0.034 | 0.897 | -0.026 | 0.922 | -0.064 | 0.806 | 0.108 | 0.682 |
| IL-2 | 0.033 | 0.897 | -0.131 | 0.611 | -0.025 | 0.921 | -0.008 | 0.975 |
| IL-4 | -0.0810 | 0.755 | -0.285 | 0.265 | 0.074 | 0.775 | -0.079 | 0.759 |
| IL-6 | 0.456 | 0.067 | 0.549 | 0.024 | -0.537 | 0.028 | 0.531 | 0.030 |
| IL-10 | -0.074 | 0.774 | 0.057 | 0.825 | 0.017 | 0.949 | 0.022 | 0.935 |
| IFN-γ | 0.152 | 0.55 | 0.285 | 0.263 | -0.223 | 0.385 | 0.271 | 0.290 |

**T****able S2.** **Correlation analysis of peripheral** **plasma cell subsets and laboratory characteristics in SFTS patients.**

Spearman correlation was used to examine the relationship between plasma cell subsets in peripheral blood and laboratory characteristics. WBC, white blood cell, RBC, red blood cell, PLT, platelet, HGB, hemoglobin, AST, aspartate aminotransferase, ALT, alanine aminotransferase, ALB, albumin, LDH, lactate dehydrogenase, cTnI, cardiac troponin I, CK-MB, creatine kinase isoenzyme, APTT, alanine aminotransferase, PT, prothrombin time, TT, thrombin time, CRP, C-reactive protein, PCT, procalcitonin.
