# Supplementary material for: Transient expansion of peripheral Lambda-expressing plasma cells represents a distinctive phenotype associated with SFTSV infection
Source: Front Immunol. 2026 Apr 24;17:1763231. doi: 10.3389/fimmu.2026.1763231 (PMC13154158; doi:10.3389/fimmu.2026.1763231)
Supplement: Supplementary file 7 [file Table3.docx]

**Table S3. Demographics and basic laboratory characteristics**

**of SFTS patients in an independent cohort.**

|  | **All patients (n=18)** | **Mild group (n=12)** | | **Severe group (n=6)** | ***P* value** |
| --- | --- | --- | --- | --- | --- |
| **Demographics feature** |  | | | | |
| Age (years) | 66 (54, 74) | 58 (54, 66) | 78 (73, 86) | | 0.001 |
| **Gender** |  | | | | |
| Male, n (%) | 8 (45%) | 3 (25) | 5 (20%) | | 0.019 |
| Female, n (%) | 10 (55%) | 9 (75%) | 1 (80%) | |  |
| **Blood routine tests** |  | | | | |
| WBC (10^9^/L) | 1.63 (1.30, 1.79) | 1.72 (1.42, 2.10) | 1.45 (1.26, 1.61) | | 0.101 |
| RBC (10^12^/L) | 4.48 (3.90, 4.86) | 4.36 (3.67, 4.72) | 4.66 (3.98, 5.07) | | 0.325 |
| Monocyte (10^9^/L) | 0.18 (0.11, 0.38) | 0.18 (0.11, 0.34) | 0.28 (0.10, 0.42) | | 0.511 |
| Lymphocyte (10^9^/L) | 0.48 (0.31, 0.69) | 0.43 (0.24, 0.65) | 0.58 (0.48, 1.82) | | 0.067 |
| PLT (10^9^/L) | 56 (34, 72) | 65 (55, 73) | 27 (17, 37) | | <0.001 |
| HGB (g/L) | 133 (116, 144) | 135 (117, 147) | 133 (113, 143) | | 0.673 |
| **Chemistry tests** |  | | | | |
| AST (U/L) | 159 (89, 1190) | 94 (61, 167) | 1256 (1188, 1791) | | <0.001 |
| ALT (U/L) | 67.75 (43.30, 196.10) | 50.25 (36.48, 68.03) | 421.80 (136.50, 618.60) | | <0.001 |
| ALB (g/L) | 31.95 (27.25, 3613) | 31.55 (24.50, 34.33) | 36.20 (29.05, 42.95) | | 0.11 |
| LDH (U/L) | 489.50 (301.50, 129340) | 389.90 (292.50, 494.80) | 1394.00 (1261.00, 1735.00) | | <0.001 |
| CK-MB (mg/L) | 4.50 (3.00, 15.50) | 4.50 (3.00, 7.75) | 13.00 (3.00, 41.00) | | 0.483 |
| **Coagulation indicators** |  | | | | |
| APTT (s) | 31.80 (28.98, 37.73) | 31.80 (29.78, 37.98) | 33.20 (26.60, 36.43) | | 0.851 |
| PT (s) | 12.75 (12.18,14.05) | 12.80 (12.13, 14.50) | 12.50 (12.08, 13.18) | | 0.482 |
| TT (s) | 20.5 (18.53, 26.53) | 19.70 (18.38, 20.92) | 29.95 (19.88, 28.90) | | 0.08 |
| D-dimer (mg/mL) | 2.28 (.86, 4.97) | 1.10 (0.40, 3.75) | 5.01 (3.07, 5.79) | | 0.02 |
| Fibrinogen (g/L) | 2.58 (2.01, 2.92) | 2.70 (2.05, 3.12) | 2.33 (1.79, 2.66) | | 0.174 |
| **Inflammation indicators** |  | | | | |
| CRP (mg/L) | 16.07 (8.10, 19.11) | 13.66 (2.04, 17.10) | 31.62 (11.03, 56.74) | | 0.039 |
| PCT (ng/mL) | 0.30 (0.04, 0.56) | 0.11 (0.02, 0.38) | 0.56 (0.40, 0.67) | | 0.007 |

Data were shown as median (interquartile range) or n (%). *P* values were calculated by two-sided Mann-Whitney *U* test or chi-square test. WBC, white blood cell, RBC, red blood cell, PLT, platelet, HGB, hemoglobin, AST, aspartate aminotransferase, ALT, alanine aminotransferase, ALB, albumin, LDH, lactate dehydrogenase, cTnI, cardiac troponin I, CK-MB, creatine kinase isoenzyme, APTT, activated partial thromboplastin time, PT, prothrombin time, TT, thrombin time, CRP, C-reactive protein, PCT, procalcitonin.
